# Attraction of the cutaneous leishmaniasis vector *Nyssomyia neivai* (Diptera: Psychodidae) to host odour components in a wind tunnel

**DOI:** 10.1186/1756-3305-5-210

**Published:** 2012-09-25

**Authors:** Mara C Pinto, Daniel P Bray, Alvaro E Eiras, Henrique P Carvalheira, Camila P Puertas

**Affiliations:** 1 Laboratório de Parasitologia, Departamento de Ciências Biológicas, Faculdade de Ciências Farmacêuticas, Universidade Estadual Julio de Mesquita Filho, 14801-902, Araraquara-SP, Brazil; 2 Chemical Ecology Group, Institute for Science and Technology in Medicine, Keele University, ST5 5BG, Keele, UK; 3 Laboratório de Ecologia Química de Insetos Vetores, Departamento de Parasitologia, Instituto de Ciências Biológicas 31270-901, Universidade Federal de Minas Gerais, Minas Gerais, Brazil

**Keywords:** Kairomone, Octenol, Lactic acid, Ammonia, Caproic acid, BG-Lure, Sandflies, Vector control, Wind tunnel

## Abstract

**Background:**

Laboratory studies of host-seeking olfactory behaviour in sandflies have largely been restricted to the American visceral leishmaniasis vector *Lutzomyia longipalpis*. In comparison, almost nothing is known about the chemical ecology of related species, which transmit American cutaneous leishmaniasis (ACL), due in part to difficulties in raising these insects in the laboratory. Understanding how ACL vectors locate their hosts will be essential to developing new vector control strategies to combat this debilitating disease.

**Methods:**

This study examined host-odour seeking behaviour of the ACL vector *Nyssomyia neivai* (Pinto) (=*Lutzomyia neivai)* using a wind tunnel olfactometer. The primary aim was to determine whether field-collected female *N. neivai* would respond to host odours in the laboratory, thereby eliminating the need to maintain colonies of these insects for behavioural experiments. Responses to two key host odour components, 1-octen-3-ol and lactic acid, and a commercially-available mosquito lure (BG-Lure™) were assessed and compared relative to an air control. We also tested whether trials could be conducted outside of the normal evening activity period of *N. neivai w*ithout impacting on fly behaviour, and whether the same flies could be used to assess baseline responses to air without affecting responses to octenol, thereby reducing the number of flies required for experiments.

**Results:**

Octenol was found to both activate host-seeking behaviour and attract female *N. neivai* in the wind tunnel, while lactic acid elicited weaker responses of activation and attractiveness under identical conditions. The BG-Lure did not activate or attract *N. neivai* under test conditions. Further experiments showed that sandfly behaviour in the wind tunnel was not affected by time of day, such that experiments need not be restricted to nocturnal hours. Moreover, using the same flies to measure both baseline responses to air and attraction to test compounds did not affect odour-seeking behaviour.

**Conclusions:**

The results of this study demonstrate that *N. neivai* taken from the field are suitable for use in laboratory olfactometer experiments. It is hoped this work will facilitate further research into chemical ecology of this species, and other ACL vectors.

## Background

Despite advances in our understanding of the chemical ecology of many important disease-transmitting hematophagic insects, and particularly mosquitoes [1], very little is known about the chemicals responsible for attracting sandfly vectors of leishmaniasis to their vertebrate hosts. Most studies of chemical ecology in sandflies have focussed on the American visceral leishmaniasis vector *Lutzomyia longipalpis* (Lutz & Neiva), in which attraction to both host odour components and male-produced pheromones have been demonstrated through laboratory olfactometer studies [2-5]. This initial work has led to field testing of synthetic *L. longipalpis* sex pheromone as a potential tool for sandfly control [6].

Of the twenty species of sandfly known or suspected to be vectors of American cutaneous leishmaniasis (ACL) [7], studies of chemical ecology have been limited to preliminary chemical analyses of potential male-produced pheromones (without evidence of behavioural attraction [8]) and field-testing of known host odour components: CO2, octenol, lactic acid, ammonia and caproic acid [9-11]. This lack of knowledge is due in part to the relative difficulty in raising these species in the laboratory compared to *L. longipalpis *[12], which prevents researchers from conducting olfactometer studies to test for behavioural responses to potentially-attractive compounds under controlled conditions. Such studies are necessary to identify chemicals which could be exploited as tools for vector control.

The aim of the present study was to determine whether it is feasible to conduct laboratory wind tunnel studies using *Nyssomyia neivai* (Pinto) (=*Lutzomyia neivai*), considered to be a vector of ACL in South America [13-15], collected directly from the field. Using field-collected insects would eliminate the need for colonizing this species prior to laboratory testing, thereby providing a means of facilitating further research into the chemical ecology of ACL vectors.

A wind tunnel was used to measure and compare responses of *N. neivai* to octenol (previously shown to attract this species in the field [11]), lactic acid, and a commercially-available mosquito lure. We also tested whether test results were affected by time of day, as in the field *L. neivai* is most active at night [16], and if the same insects could be used to measure both baseline responses to air and activation/attraction to test stimuli, thereby reducing the number of insects required for each experiment.

## Methods

### Field collection and laboratory maintenance of insects

Insects were collected during September and October (21° 35′13′S 48° 04′15′W - 12 hours natural light per day) using automatic light traps and manual aspiration from a previously studied area where 99.9% of sandflies were identified as *N. neivai *[11]. Following arrival at the laboratory, sandflies were maintained in netting cages at 26 ± 1°C, 80–90% humidity, 12:12 (L:D) photoperiod with access to 30% sucrose solution. The laboratory where sand flies were kept and the wind tunnel experiments were carried out have fluorescent lights, and no access to natural light sources (e.g. windows). The fluorescent lights are controlled by a timer that switched on at 07:00 and switched off at 19:00.

All tests started on the day after sandfly capture and were conducted within three consecutive days. Following experiments, insects in both test and control groups were killed in 70% ethanol and stored for identification, following the classification of Galati [17,18] and the nomenclature of Young and Duncan [19], and female abdomens inspected for the presence/absence of a blood-meal and eggs.

### Wind tunnel apparatus

**Figure 1:**
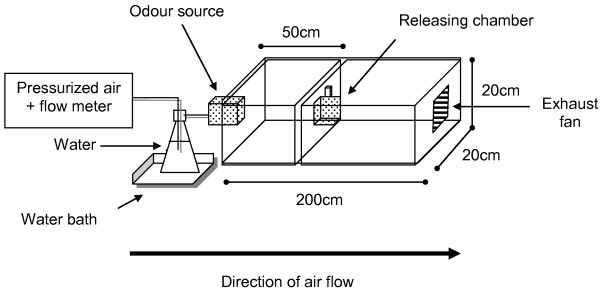
**Wind tunnel olfactometer.** Wind tunnel apparatus used to assess responses of female *N. neivai* to host odour components. Humidified compressed air flowed through the wind tunnel from left to right. Sandflies which left the releasing chamber were recorded as having been activated by the odour source. Sandflies which travelled to the end of the wind tunnel were recorded as having been attracted by the odour source.

All bioassays were conducted in a transparent acrylic wind tunnel (length 200 cm, width 20 cm, height 20 cm; Figure 1). Air flow was obtained from a zero grade air cylinder, humidified using a water bath (36°C) prior to entry through the end of the tunnel where odour sources would be presented. Air flow was set at 500 ml min^-1^ using a rotameter. Conditions within the wind tunnel were recorded as 24 ± 1°C and 70-75% relative humidity. In order to maintain a constant flow of air in the tunnel, and prevent the build-up of test compounds within the apparatus, air was allowed to exit the tunnel at the opposite end to entry through an exhaust (Figure 1).

### Bioassay protocol

Groups of three female *N. neivai* were placed inside a releasing chamber (Figure 1) for 30 min to acclimatize prior to each test. The chamber was then placed inside the wind tunnel 50 cm downwind from the air intake and odour source, and the sandflies released by opening the chamber door. Each trial lasted 2 min, with activation (number of sandflies leaving the releasing chamber) and attraction (number of sandflies reaching the odour source) recorded for each group.

As a negative control, baseline responses to air flow alone were always ascertained prior to the introduction of each test chemical. The host odour component 1-octen-3-ol (98.0% pure by gas chromatography; Aldrich Chemical, Milwaukee, WI, hereafter referred to as octenol), previously shown to attract *N. neivai* in the field [11], was used as a positive control, and to measure sandfly responses under different conditions. Except where stated, different individual sandflies were used for measuring responses to test and control stimuli. All experiments were conducted under white fluorescent light.

### Experiment 1. Can field-caught *N. neivai* be used to measure olfactory responses in the wind tunnel?

The first experiment was conducted to determine the viability of using insects taken directly from the field in bioassays, by measuring their responses to a known attractive host odour component. The test chemical, octenol, was released from micro-reaction vials following the methodology of Van Essen *et al*. [20]: 1 ml was placed into each 5 ml tube, with two pieces of string in contact with the octenol passing through the tube lid via two small holes, leaving 4 cm of each length exposed outside the tube. Test vials were then placed into the wind tunnel at the air intake. Octenol release rate was determined by weighing tubes before and after experiments.

Prior to presentation of octenol, a control group of insects (N = 50) were exposed to air flow only to ascertain baseline activation and attraction responses in the wind tunnel. Responses to octenol at 28 mg h^-1^ were then measured using a separate group of insects (N = 50).

### Experiment 2. Can the same insects be used to measure responses to control and test stimuli?

The aim of this experiment was to determine whether the same group of *N. neivai* could be used to measure baseline responses to the air control without influencing activation/attraction to the test stimuli, thereby reducing the total number of wild-caught flies needed for wind tunnel experiments.

Responses of two groups of flies to octenol (32 mg h^-1^) were compared: those with no prior test experience (N = 50), and those which had previously been exposed to the control stimuli (air) (N = 40) approximately 2 h beforehand.

### Experiment 3. Does time of day influence *N. neivai* responses in the wind tunnel?

To determine whether the sandflies response to host odour could be measured in the wind tunnel outside of their normal nocturnal activity peak, responses of female *N. neivai* to octenol were measured and compared in experiments conducted between 09:00–12:00 and 15:00–18:00 and 19:00–22:00. Octenol was released by placing 200 μl onto filter paper (4x4cm) in the entrance of the wind tunnel.

For each time period, baseline responses to air (N = 30) and then octenol (N = 30) were measured using the same group of flies.

### Experiment 4. Is *N. neivai* attracted to lactic acid in the wind tunnel?

This test measured sandfly responses to lactic acid, also an important host odour component, in comparison to octenol under identical laboratory conditions. Responses to L-lactic acid (85% P.A., Synth), octenol, and the air control were measured using three separate groups of flies (N = 30). Lactic acid and octenol were released by placing 100 μl of the required test chemical onto filter paper (4x4 cm) in the entrance of the wind tunnel.

### Experiment 5. Is *N. neivai* attracted to the BG-lure?

The BG-Lure™ (Biogents AG, Regensburg, Germany) is a commercially available lure used for the capture of *Aedes aegypti* through release of a mixture of human odour components: lactic acid, ammonia and caproic acid [21]. This experiment tested whether this same lure could activate and attract female *N. neivai* in the wind tunnel, and ascertain its relative attractiveness compared to octenol and the air control.

Activation and attraction behaviours of flies (N = 65) were measured in response to either a BG-Lure placed at the end of the wind tunnel, octenol released from a micro-reaction vial at 15 mg h^-1^ (N = 50) and the air control (N = 50). Different flies were used for each test.

### Statistical analysis

Pearson’s chi-square tests were used to test for a difference in number of flies activated and attracted by different test stimuli and the air control. Fisher’s exact test was used instead where expected values in the contingency table were less than five [22], as indicated in the text.

The same methodology was used to test whether prior exposure to the air control influenced number of flies responding to octenol and air, and to compare responses at different times of the day.

Where negative results are reported (i.e. no statistical difference found), post-hoc power analysis was performed to determine the power of the experiment to detect a medium-sized effect of 0.3 [23] with the sample sizes used. All analyses were performed using R [24,25].

The responses of sandflies to octenol were compared between experiments in a post-hoc analysis, investigating the possibility of an effect of odour-release method on *N. neivai* behaviour in the wind tunnel. Chi square tests were used to test whether there was a significant difference in the proportion of sandflies both activated and attracted by the three release-methods used (micro-reaction vial loaded with 1 ml octenol, filter paper loaded with 100 μl octenol, filter paper loaded with 200 μl octenol).

## Results

### Collected sandflies

All 495 field-collected sandflies were identified as *N. neivai*. Only six females were found to contain blood meals, with four gravid with eggs. These flies did not appear to show any consistently different responses from non-gravid or non-bloodfed flies in the wind tunnel.

### Experiment 1

Significantly more female *N. neivai* were activated by the presence of octenol in the wind tunnel than the air control (air 2/50, octenol 34/50, Χ^2^ = 41.7, df = 1, P < 0.001). Similarly, octenol attracted more females to the end of the wind tunnel than air flow alone (air 1/50, octenol 25/50, Χ^2^ = 27.5, df = 1, P < 0.001). This demonstrates both that octenol is attractive to female *N. neivai* in the wind tunnel, and that flies taken directly from the field can be used in laboratory olfactometer experiments.

### Experiment 2

There was no significant effect of prior exposure to the control stimulus on number of sandflies activated by octenol (no prior exposure 38/50, prior exposure 29/40, Χ^2^ = 0.02, df = 1, NS, power = 0.81). Similarly, there was no effect of prior exposure to air on numbers attracted to octenol (no prior exposure 26/50, prior exposure 22/40, Χ^2^ = 0.005, df = 1, NS, power = 0.81).

As in Experiment 1, in total a greater proportion of flies were both activated by octenol than the air-only control (octenol 67/90, air 2/50, Χ^2^ = 61.0, df = 1, P < 0.001), and attracted to octenol compared to the air control (octenol 48/90, air 1/50, Χ^2^ = 35.0, df = 1, P < 0.001).

### Experiment 3

**Figure 2:**
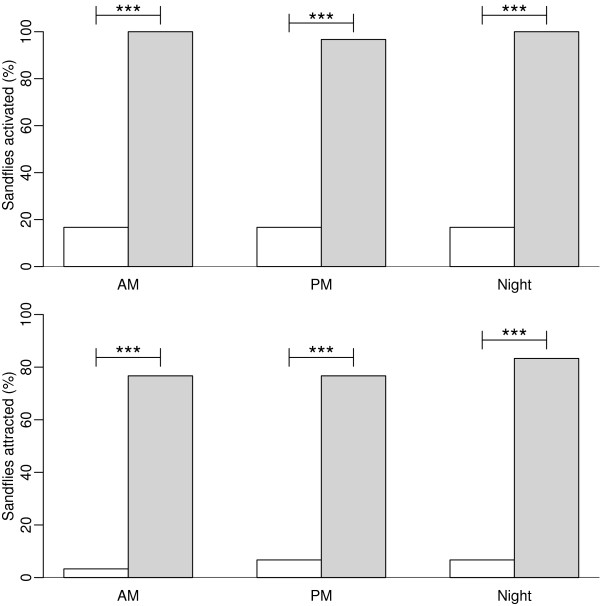
***N. neivai *responses to air and octenol at different times of the day.** Percentage of female *N. neivai* (N = 30) activated (top graph) and attracted (lower graph) by air (white bars), and octenol (grey bars) in the wind tunnel (Experiment 3). * P < 0.05, ** P < 0.01, *** P < 0.001.

Baseline proportions of *N. neivai* activated and attracted by air were not affected by the time of day at which experiments took place (activation: Χ^2^ = 0, df = 2, NS; attraction: df = 2, NS (Fisher’s exact test (approximation for 2x3 table); power = 0.72; Figure 2).

Similarly, responses to octenol also did not differ significantly according to time of day at which the experiments were performed (activation: Χ^2^ = 2.0, df = 2, NS; attraction: Χ^2^ = 0.53, df = 2, NS, power = 0.72; Figure 2).

Octenol activated a greater proportion of sandflies than air in the morning (octenol 30/30, air 5/30, X^2^ = 39.5, df = 1, P < 0.001), afternoon (octenol 29/30, air 5/30, X^2^ = 35.9, df = 1, P < 0.001) and evening (octenol 30/30, air 5/30, X^2^ = 39.5, df = 1, P < 0.001; Figure 2). Similarly, octenol attracted more sandflies than air in the morning (octenol 23/30, air 1/30, X^2^ = 30.6, df = 1, P < 0.001), afternoon (octenol 23/30, air 2/30, X^2^ = 27.4, df = 1, P <0.001) and evening (octenol 25/30, air 2/30, X^2^ = 32.6 df = 1, P <0.001; Figure 2), confirming that time of day had no detectable effect on *N. neivai* responses in the wind tunnel.

### Experiment 4

**Figure 3:**
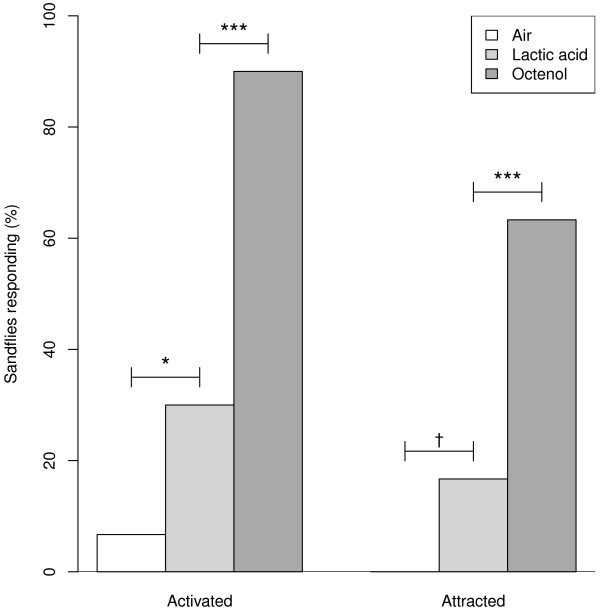
***N. neivai *responses to air, lactic acid and octenol.** Percentage of female *N. neivai* activated and attracted by air (N = 30), lactic acid (N = 30) and octenol (N = 30) in the wind tunnel (Experiment 4). * P < 0.05, ** P < 0.01, *** P < 0.001, † P = 0.052.

A higher proportion of females were activated by lactic acid in the wind tunnel compared to the air control (lactic acid 9/30, air 2/30, Χ^2^ = 4.01, df = 1, P < 0.05; Figure 3). Lactic acid also elicited an attractive response, which bordered on significance (lactic acid 5/30, air 0/30, df = 1, P = 0.052) (Fisher’s exact test). However, responses to lactic acid were relatively weak compared to octenol, which both activated (27/30, Χ^2^ = 20.1, df = 1, P < 0.001; Figure 3) and attracted (19/30, Χ^2^ = 11.7, df = 1, P < 0.001; Figure 3) a higher proportion of *N. neivai.*

### Experiment 5

**Figure 4:**
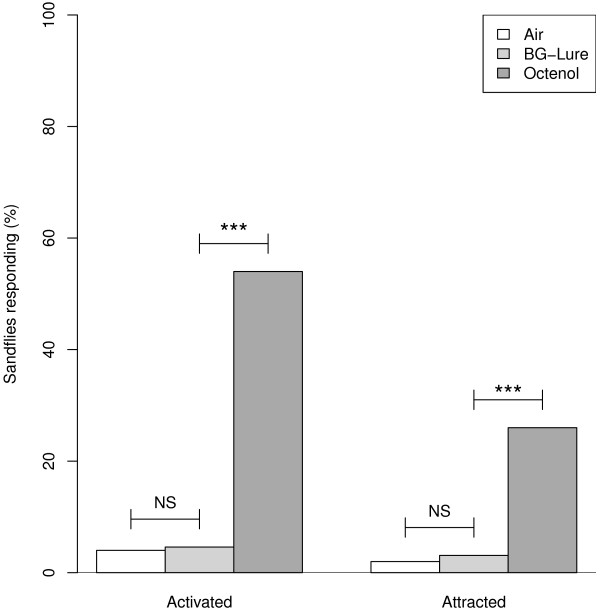
***N. neivai *responses to air, BG-lure and octenol.** Percentage of female *N. neivai* activated and attracted by air (N = 50), BG-Lure (N = 65) and octenol (N = 50) in the wind tunnel (Experiment 5). * P < 0.05, ** P < 0.01, *** P < 0.001, NS Not Significant.

The BG-Lure did not elicit significantly greater responses from *N. neivai* than the air control (activation, BG-Lure 3/65, air 2/50, df = 1, NS (Fisher’s exact test); attraction, BG-Lure 2/65, air 1/65, df =1, NS (Fisher’s exact test); power = 0.89; Figure 4). In the same experiment, octenol activated (27/50, Χ^2^ = 33.2, df = 1, P < 0.001; Figure 4) and attracted (13/50, Χ^2^ = 11.1, df = 1, P < 0.001, Figure 4) a significantly greater proportion of sandflies then the BG-Lure.

### Sandfly responses to octenol under different conditions

In a post-hoc analysis of sandfly responses between experiments, a significant overall difference was found between tests using different methods of octenol release in terms of both the number of sandflies activated (Χ^2^ = 19.6, df = 2, P < 0.001) and attracted (Χ^2^ = 13.9, df = 2, P < 0.001; Table 1).

**Table 1:** **Proportion of female *N. neivai *activated and attracted in the wind tunnel in response to different methods of octenol release**

**Experiment**	**Release method**	**Octenol loaded (released)**	**Proportion**	**Proportion**
			**Activated (%)**	**Attracted (%)**
1	Micro-reaction vial	1 ml (28 mg h^-1^)	34/50 (68) ^a^	25/50 (50) ^a^
2	Micro-reaction vial	1 ml (32 mg h^-1^)	38/50 (76) ^a^	26/50 (52) ^a^
3	Filter paper	200 μl	30/30 (100) ^b^	23/30 (83) ^b^
4	Filter paper	100 μl	27/30 (90) ^b^	19/30 (63) ^b^
5	Micro-reaction vial	1 ml (15 mg h^-1^)	27/50 (54) ^a^	13/50 (26) ^a^

^a b^ Different letters within a column indicate significant differences in response towards micro-reaction vials and filter paper.

In subsequent analyses, no difference was found between experiments using filter paper loaded with 200 μl or 100 μl of octenol in the number of sandflies activated (df = 1, NS (Fisher’s exact test) or attracted (Χ^2^ = 0.71, df = 1, NS)). However, more *N. neivai* were both activated (Χ^2^ = 17.4, df = 1, P < 0.001) and attracted (Χ^2^ = 11.7, df = 1, P < 0.001) overall in experiments using filter paper as a release method than those employing micro-reaction vials.

## Discussion

Experiments using blood-feeding insects reared under identical laboratory conditions can control for factors such as physiological status, which might influence host-seeking behaviour [26]. However, for *N. neivai*, as for many other species of sandfly, laboratory rearing is difficult to maintain over more than a few generations [27], with the small number of insects produced being a limiting factor in conducting behavioural studies.

The results of this study demonstrate that, even though sandflies were taken from the field, and were of unknown age and nutritional status, female *N. neivai* showed consistently higher activation and attraction responses to 1-octen-3-ol in laboratory bioassays than an air control. In nature, octenol is released from a diverse range of sources, including fungi [28] and vertebrate breath [29,30]. This semiochemical, previously identified as attractive to *N. neivai* in field studies [11], also proved to be an excellent positive control for wind tunnel experiments which seek to assess responses to other potential sandfly attractants.

Often, the number of sandflies captured from the field is not sufficient to conduct all the necessary experiments in planned behavioural studies. The results of the current work showed that groups of *N. neivai* used to assess baseline responses to air flow in the wind tunnel can be reused without influencing the number of flies responding to octenol, as compared to a separate group of flies with no test experience. More studies are required to determine whether the same might be generally applicable following exposure to other semiochemicals, or if any form of learning or habituation occurs in *N. neivai*, as reported for mosquitoes [31] and other species of sandfly [32,33].

The time at which experiments are conducted is another potential source of variation in sandfly responses in laboratory bioassays: most insects follow daily cycles in activity, in part governed by endogenous circadian rhythms [34]. In nature, *N. neivai* is typically most active during nocturnal hours [16], but no previous study has examined how time of day might affect *N. neivai* responses to kairomones under laboratory conditions. Bioassays with *L. longipalpis* have been successfully performed at different times of the day (e.g. the start of the scotophase [5] and 0900–1300 [35]). The results here showed no difference in *N. neivai* responses to octenol during different parts of the day, suggesting that laboratory experiments with field collected insects need not be restricted to night and evening. However, similar experiments with other compounds should be performed to determine the general applicability of this observation. We should also make clear that all of the bioassays conducted for experiment 3 were carried out during the same 24 h period, with the lights on even during the night session (19:00–22:00), such that the insects had no exposure to a scotophase before the bioassay. It seems therefore that under the described conditions there was no effect of any underlying circadian rhythm on *N. neivai* host odour-seeking behaviour: previous work in *L. longipalpis* suggests that such rhythms in locomotory behaviour do occur in sandflies, and may be modulated by blood intake [36].

Lactic acid elicited activation responses in the wind tunnel, but was only weakly attractive: octenol both activated a greater number of *N. neivai*, and was significantly more attractive. A component of human breath [37], attraction to lactic acid has been well studied in mosquitoes, and in general is more attractive when presented synergistically in combination with other host odour components, such as CO2, carboxylic acid, ammonia or acetone [38-42]. In sandflies, attraction to lactic acid has only previously been assessed in the field, and was not found to be attractive to *L. longipalpis* or *N. intermedia *[9]. More studies are needed to assess sandfly attraction to lactic acid when presented with other host odour components, including octenol.

The BG-Lure, which releases a mixture of lactic acid, ammonia and caproic acid, was not attractive to *N. neivai*. This lure was developed to mimic human odour and has been shown to attract *A. aegypti*[21]. Interestingly, while lactic acid alone did attract a small number of sandflies, there was no response whatsoever to the BG-Lure. Potentially, this might be because either the amount of lactic acid released is too small to evoke even a minimal response, or that the other chemicals released by the lure have some masking or repellent effect.

This study made use of two different methods of releasing test chemicals within the wind tunnel. Experiments 1, 2 and 5 were performed using micro-reaction vials, following the same methodology that has been previously used in the field [10]. While octenol released from these vials did activate and attract sandflies in the wind tunnel, measurements of the weight of these vials before and after experiments suggest considerable variation may exist in their individual release rates, even when two vials are prepared and set to release under identical conditions. One potential source of this variation could be the amount of string in contact with the octenol in each vial.

Experiments 3 and 4 (performed at a later date) made use of filter paper as a release substrate. The volume of octenol was not reapplied onto filter paper during the bioassays because the responses of the sandflies did not change along the test period. Results of post-hoc analysis comparing between experiments suggest that a greater proportion of *N. neivai* were both activated and attracted by octenol released from the filter paper than from the vials, perhaps indicating a faster release rate from filter paper, and hence a greater response. However, this hypothesis requires stricter testing in order to draw firm conclusions.

## Conclusions

The results of this study demonstrate that it is possible to use wild-caught *N. neivai* in wind tunnel experiments to assess responses to host odour, and highlight the greater attractiveness of octenol than lactic acid under controlled conditions. It is hoped that these results will facilitate further studies into host-seeking behaviour of both *N. neivai* and other vectors of cutaneous leishmaniasis, with the aim of developing new tools to control this debilitating, neglected disease.

## Competing interests

The authors declare that they have no competing interests.

## Authors’ contributions

MP conceived and executed the study and drafted the manuscript. DB provided additional statistical analysis and figures and contributed to the manuscript. AE provided the BG-Lure and contributed to the manuscript. HC and CP conducted field captures of sandflies and bioassay experiments. All authors read and approved the final manuscript.
